# Modulatory effect of porous silicon water-formulated catechin on gut microbiome in chronic unpredictable mild stress-induced dementia in a rat model

**DOI:** 10.3389/fphar.2026.1778580

**Published:** 2026-06-05

**Authors:** Sulaiman Mohammed Alnasser, Srinidhi Ravikumar, Saravanan Jayaraman, Divakar Selvaraj, Venkatesh Gunasekaran

**Affiliations:** 1 Department of Pharmacology and Toxicology, College of Pharmacy, Qassim University, Qassim, Saudi Arabia; 2 Department of Pharmacology, KMCH College of Pharmacy, The Tamil Nadu Dr. M. G. R. Medical University, Chennai, India; 3 Department of Pharmacology, JSS College of Pharmacy, JSS Academy of Higher Education & Research, Ooty, Tamil Nadu, India; 4 Prime Laboratory Animal Breeding & Research Centre, Department of Pharmacology, Prime College of Pharmacy, Palakkad, Kerala, India; 5 Department of Pharmacology, PSG College of Pharmacy, The Tamil Nadu Dr. M.G.R. Medical University, Chennai, India

**Keywords:** catechin, chronic unpredictable stress, dementia, glutamate, gut microbiome, porous silicon

## Abstract

Stress-induced dysbiosis exacerbates mental health by modulating the nervous system and gut permeability. In this study, we investigate the therapeutic potential of porous silicon water-mixed catechin in alleviating chronic stress-induced dementia in rats. In a 28-day study, chronic unpredictable mild stress (CUMS)-induced rats were treated with *Lactobacillus acidophilus* (2.5 × 10^9 CFU, p.o), porous silicon water (7 mg/kg, p.o), catechin (30 mg/kg, p.o), and porous silicon water-mixed catechin (PSC) (7 mg and 30 mg/kg, p.o). The effect of porous silicon water-mixed catechin was evaluated through behavioral studies, plasma acetylcholinesterase activity, plasma glutamate, brain reactive oxygen species (ROS), brain endogenous anti-oxidant enzymes, metagenomics analysis, and histological examination of the prefrontal cortex and hippocampus. Administration of PSC significantly improved spatial learning and memory by reducing escape latency time and increased exploratory behavior in the open platform. PSC significantly inhibited acetylcholinesterase enzyme activity and restored endogenous antioxidants such as superoxide dismutase (SOD), catalase (CAT), and glutathione reductase (GSH) while reducing lipid peroxidation (LPO) compared to the CUMS group. In addition, PSC decreased the brain ROS levels, as determined by a fluorescence assay, and reduced plasma glutamate levels. 16S rRNA V3–V4 metagenomic analysis revealed a significant increase in microbial diversity (Shannon index: 7.52), microbial richness (Chao1 index: 1059.41), β-diversity index, and overall taxonomic abundance in treated rats. CUMS-induced morphological alterations in the hippocampus and prefrontal cortex were significantly improved following PSC administration. In the present study, Pearson correlation coefficient (r) demonstrates an association between gut microbial abundance and AChE activity. Hence, it has been concluded that PSC treatment may significantly modulate the gut microbiome and improve cognition in chronic unpredictable mild stress-induced dementia.

## Introduction

The gut microbiota, comprising diverse microbial communities, is crucial for maintaining host metabolic equilibrium. The gut–brain axis has been explored extensively since the National Institute of Mental Health (NIMH) initiative in 2013 (Jašarević et al., 2015; Ogbonnaya et al., 2015). Bidirectional communication between the gut microbiota and the brain is mediated by three major pathways: vagus nerve, enteric nervous system, and cross-talk between pro-inflammatory cytokines, hormones, and neurotransmitters (Agirman and Hsiao, 2021). Stress-induced dysbiosis can exacerbate mental health disorders by modulating the inflammatory pathway and gut permeability (Jiang et al., 2015; Li et al., 2019; Vogt et al., 2017), resulting in a “leaky gut” and making way for the translocation of pathogens into the portal and systemic circulation. This contributes to redox mechanism and neuroinflammation in CNS disorders (Fasano, 2020; Theoharides et al., 2013).

Regulation of neuroactive metabolites, immune modulators, and intestinal barrier function by gut microbes could influence cognitive function (Kearns, 2024). Alzheimer’s patients with Aβ-positive condition are directly associated with imbalanced microbial abundance of *Protococcus*/*Bacillus subtilis* and *E. coli/Shigella spp.* in the gut (Liu et al., 2022). This leads to elevated concentrations of pro-inflammatory cytokines in the brain. Furthermore, these microbes release lipopolysaccharides (LPS) that triggers microglial cell activation, which induces neuronal cell injury leading to cognitive dysfunction (Yao et al., 2022). Similarly, abnormal gut microbiota stimulates the vagus nerve and the hypothalamic–pituitary–adrenal (HPA) axis during stress, affecting cognition and mood regulation (Carabotti et al., 2015). Recent therapies for cognitive impairment, such as probiotics, prebiotics, or fecal microbial transplantation, focus primarily on targeting the gut microbiota (Zhao et al., 2021).

Chronic unpredictable mild stress (CUMS) is a valid model to examine depression, learning, and memory-related activities. Studies have demonstrated that long-term exposure to unpredictable stress significantly interrupts brain biochemical reactions that could impair neuronal growth, learning, and memory processes compared to predictable stress. Furthermore, it activates the HPA axis-mediated glucocorticoid release, which could influence the short-term and long-term cognitive function (Afshari et al., 2014). Oversecretion of corticosterone or chronic stress exposure causes changes in brain morphology, neurogenesis, and neuronal cell death, which are causal mechanisms of stress-induced learning and memory impairment (Conrad, 2010). CUMS not only affects the neuronal and psychological functions but also influences the gut metabolic reaction, intestinal barrier function, visceral activities, and alteration of fecal microbiota in rodents and humans (Li et al., 2023; Xu et al., 2023).

Short-chain fatty acids secreted by the microbes act as neuro-endocrine signal mediators in the enteric nervous system and can influence the gut–brain regulation, impacting the anatomical and functional role of tight junction proteins (Varatharaj and Galea, 2017). Beneficially altering the composition of gut microbiota can mitigate oxidative stress and inflammation, offering potential therapeutic outcomes (Cattaneo et al., 2017; Zhuang et al., 2018). Studies have reported that hydrogen and hydrogen-rich water are absorbed and distributed widely in the body. This has gained significant attention due to its remarkable ability to eliminate hydroxyl radicals and promising beneficial effects in preventing various diseases, including cancer, Parkinson’s disease, Alzheimer’s disease, diabetes, obesity, atopic dermatitis, and cutaneous senility (Tabassum et al., 2020).

Silicon powder, a non-toxic biocompatible and biodegradable substance, plays a promising role in generating hydrogen within the body fluids that potentially offers a safer alternative to inhaling hydrogen–oxygen mixture (Anglin et al., 2008). Porous silicon is used to generate hydrogen gas, which is equivalent to approximately 1,600 mL of hydrogen gas per gram of silicon (Manilov, 2016). Moreover, silicon with its porous structure is potentially used in the biomedical field due to its biodegradability, non-cytotoxic activity, and non-immunogenic behavior, along with better adaptability (Ben Belgacem et al., 2018; Zare et al., 2021). Catechin abundant in green tea and other food substances protects cells and tissues from oxidative stress, thereby promoting overall health and longevity. They are also linked to various health benefits, including neuroprotection in neurodegenerative disorders (Bernatoniene and Kopustinskiene, 2018; Du et al., 2019; Xiang X et al., 2016). Hence, we hypothesize that the infusion of catechin-mixed porous silicon water offers a comprehensive approach in restoring gut microbial balance and mitigating neural dysregulation associated with chronic stress. This innovative intervention appears promising for addressing and balancing the intricate interplay between gut health and cognitive function, thereby offering potential therapeutic benefits for individuals at risk of dementia condition.

## Materials and methods

### Animal and study design

Male Wistar rats (150–180 gm) were sourced from the Biogen Laboratory Animal Facility in Bangalore (Reg. No: 971/bc/06- CPCSEA). A group of six animals were housed in a cage. Experimental procedures were conducted with approval from the Committee for the Purpose of Control and Supervision of Experiments on Animals (CPCSEA)—KMCRET/ReRc/M Pharm/79/2023. The animals were randomly selected and grouped (n = 6) as follows: Group 1 served as the normal control, receiving 0.9% w/v normal saline orally (p.o); Group 2 was subjected to CUMS; Group 3 received CUMS along with *Lactobacillus acidophilus* (2.5 × 10^9 CFU, p.o); Group 4 received CUMS along with porous silicon water at doses of 7 mg/kg; Group 5 received CUMS along with catechin 30 mg/kg (p.o), and Group 6 received CUMS along with porous silicon (7 mg/kg) and catechin (30 mg/kg) orally.

### Preparation of porous silicon water mixed with catechin

**FIGURE 1:**
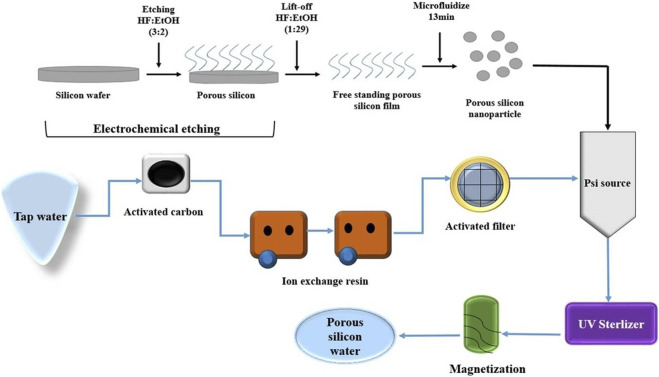
Preparation of porous silicon water generated using the electrochemical etching method.

Porous silicon water generated via electrochemical etching mediated anodization of crystalline silicon wafers in a hydrofluoric acid (HF) solution. The desired thickness of the silicon wafer was processed through fabrication techniques. Then, it was subjected to anodization by applying electric current in an electrolyte solution to scratch pores onto the silicon surface. Thereafter, the silicon layer was soaked with deionized water and cautiously dried and cleaned. Then, the wafers were anodized to etch pores, followed by refinement and rinsing (Figure 1). Shortly after, 7 mg of silicon powder was loaded into a column, through which tap water was passed after purification via activated carbon, ion exchange resin, and activated filter columns. The treated water underwent UV sterilization, magnetization, and was then assayed at 812 nm using a UV–visible spectrophotometer following the molybdosilicate method for the estimation of concentration. Catechin was mixed with 5 mL of prepared porous silicon water and administered orally (Anglin et al., 2008; Wu et al., 2021).

### Behavioral study

#### Chronic unpredictable mild stress induction protocol

**FIGURE 2:**
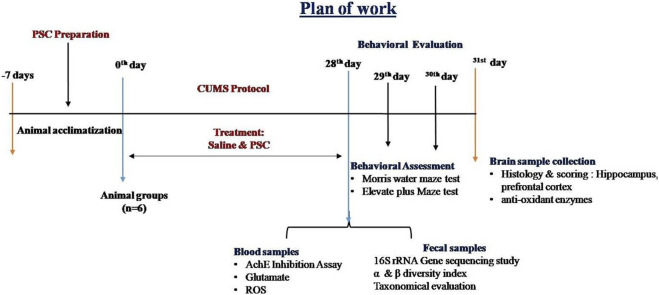
Timeline protocol for the chronic unpredictable mild stress-induced dementia model in rats treated with silicon, catechin, and PSC.

After a 7-day acclimatization period, before rats were subjected to the CUMS, the animals were evaluated randomly in the sucrose preference test to validate the animal model. Various stressors were then administered for 4 weeks. Each day, a different stressor was applied in a random order. Control rats were monitored separately and kept isolated from the stressed animals. The procedure for the induction of stress is as follows: on Day 1, rats were deprived of water for 24 h and subjected to tail pinching for 1 min, repeated twice at a 6-h interval; on day 2, the rats were housed in a crowded environment (10 rats per cage for 12 h). On day 3, the rats were housed in a continuously illuminated and isolated environment throughout the day and night. The rats were deprived of food for 24 h on day 4. The rats were housed in a cage tilted at 45° angle for 12 h on day 5. On day 6, the rats were subjected to a novel odor, along with limited food (45 g). The rats were exposed to soiled bedding (150 mL water per cage for 12 h) on day 7. This protocol was repeated during the second, third, and fourth weeks of the experiment, maintaining consistency alongside drug treatment for 28 days (Figure 2) (Veintramuthu et al., 2018).

### Morris water maze experiment

Morris water maze (MWM) from Inco Manufacturers evaluates spatial learning in rodents, relying on distal cues for navigation. Rats navigated from start points around a circular pool to locate a submerged platform. The test, conducted in a black circular pool (160 cm diameter, 80 cm height) filled with water (40 cm depth, 21 °C ± 2 °C), features a platform (10 cm diameter) submerged 1.5 cm below the water surface in one quadrant. Trials were conducted in a dimly lit room with visual cues. Rats received four trials daily, with the platform’s position unchanged. Each trial lasted for 60 s, with rats gently placed in the water and allowed to find the platform. Failure results in gentle guidance to the platform. The measurement of escape latency on day 6 is considered acquisition or learning. Rats underwent 6 days of training, with water stirred between trials to erase olfactory traces (El-Marasy et al., 2012).

### Elevate plus maze test

Elevate plus maze (EPM) comprises four arms: two open and two enclosed arms. Each arm measures 50 cm in length by 10 cm in width, with the enclosed arms featuring 30-cm high walls. Rats were placed at the intersection of the arms and observed for 5 min. The number of entries and the time spent in the open and closed arms were recorded. Post-trial, rodents were gently removed, and the maze was sanitized for subsequent trials (Walf and Frye, 2007).

### *In vitro* acetylcholinesterase inhibition assay

Ellman’s procedure was used to measure cholinesterase activity. The acetylthiocholine substrate is hydrolyzed by acetylcholinesterase, generating thiocholine, which reacts with Ellman’s reagent (DTNB). This reaction produces detectable products at 412 nm. In the assay, 0.4 mL of blood plasma is mixed with 2.6 mL of phosphate buffer and 100 μL of DTNB reagent. Then, 20 μL of the acetylthiocholine iodide substrate is added, and the change in optical density at 412 nm is measured every 2 min for 10 min. AChE activity is calculated using the formula: R = 5.74 × 10^−4^ × ΔA/C, where R represents the rate of enzymatic activity in μmol/min/mg of proteins, ΔA is the change in the absorbance per minute, and C is the concentration of tissue homogenate (Ellman et al., 1961).

### Excitatory amino acid estimation by HPTLC analysis

Glutamate estimation in plasma using the HPTLC method involves several steps. First, 0.1 N HCl prepared with 80% ethanol is used to dissolve L-glutamic acid for the standard solution. 0.2% ninhydrin solution is prepared by dissolving 200 mg of ninhydrin in 100 ml of n-butanol. HPTLC plates are prewashed with methanol before sample injection. Standard solution (20 μL) and plasma samples (20 μL) are spotted on the plates using a Linomat V applicator. The mobile phase, consisting of n-butanol, glacial acetic acid, and water (60:15:25 v/v) was allowed to saturate the chamber for 15 min before the sample-spotted plates were run to a distance of 8 cm in the chamber. After drying, the plates were sprayed with ninhydrin solution and scanned using the CAMAG TLC scanner III at a wavelength of 550 nm. Chromatographic conditions included the use of HPTLC silica gel GF254 plates, an ascending mode of development, and room temperature, with 55%–65% relative humidity (Venkatesh et al., 2019).

### Brain ROS estimation using the 2′,7′-dichlorodihydrofluorescein diacetate assay

Brain tissue was homogenized with PBS buffer solution at pH 7.4, and then, the homogenate was centrifuged at 10,000 rpm/15 min. Thereafter, the supernatant liquid was collected. A 100 μL aliquot of treated and untreated brain samples was mixed with 100 μL of a 10 μM 2′,7′-dichlorodihydrofluorescein diacetate (DCFH-DA) solution, protected from light, and incubated at 37 °C for 25–30 min to allow oxidation of DCFH to DCF, according to the protocol. The intensity of 2′,7′-dichlorofluorescin was measured using a fluorescence microplate reader at an excitation wavelength of 495 nm and an emission wavelength of 525 nm (Cathcart et al., 1983).

### Estimation of endogenous anti-oxidant enzymes in the rat brain

Brain tissue weighing 100 mg was homogenized in 10 mL of Tris hydrochloric acid buffer (0.5 M; pH 7.4) at 4 °C. Following centrifugation, the supernatant was collected for the assay. The catalase (CAT) assay measures the continual decrease in H_2_O_2_ absorbance in the presence of CAT. Tissue homogenate (50 μL) was mixed with PB-H_2_O_2_ solution (3 mL), and absorbance was measured at 240 nm. For superoxide dismutase (SOD) activity, tissue homogenate (50 μL) was added to sodium carbonate buffer (1.85 mL), followed by the direct addition of epinephrine (100 μL) into the cuvette. The reaction was then measured at 295 nm. The presence of SOD (U/mg of protein) was derived from a reference plot using the photometric technique. For glutathione reductase (GSH), tissue homogenate (500 μL) was treated with TCA solution (500 μL) and centrifuged to obtain the supernatant. The supernatant was then mixed with PBS (3 mL) and DTNB (500 μL) and incubated for 10 min at 27 °C. The absorbance of the resulting yellow chromogen at 412 nm is directly proportional to GSH concentration. For lipid peroxidation (LPO), malondialdehyde served as a marker of LPO. Brain homogenate (1 mL) was mixed with TCA-TBA-HCl reagent (2 mL) and placed for 15 min in a water bath. After cooling and centrifugation, the absorbance of the supernatant was measured at 532 nm. Malondialdehyde concentration was calculated using the molar extinction coefficient of 1.56 × 10^5^ M^−1^cm^−1^ (Khan et al., 2012).

### Fecal sample analysis

In 16S metagenomics, genomic material was sequenced directly from fecal samples (N = 6), targeting hyper-variable regions such as V3, V4, and V3–V4 amplicons of the 16S ribosomal gene. Fecal samples were taken, and the library preparation for V3–V4 amplicon sequencing involved several enzymatic steps using the NEBNext Ultra DNA Library preparation kit. These libraries were quantified and loaded onto the cBot for cluster generation and sequencing, resulting in 2 × 250 bp sequence reads. Subsequently, sequencing was carried out on the Illumina NextSeq 2000/1000 to produce 2 × 300 bp reads per sample. Quality control ensured that up to 75% of bases reached a Q30 value. These quenched data were processed to generate FASTQ files, which were uploaded to the FTP server for download. Bioinformatics analysis involved quality trimming, amplicon detection, de-replication, and chimera removal. Sequences were then grouped into operational taxonomic units (OTUs) and classified using the RDP classifier against the Green Gene database. Finally, α- and β-diversity metrics were calculated to assess within-sample and between-sample microbial diversity, providing insights into sample microbial composition and community structure (Yang et al., 2022).

### KEGG pathway prediction analysis

The molecular targets for catechin were collected from various databases, including PubChem, Binding DB, and the Similarity Ensemble Approach. A total of 63 gene targets were identified from these sources. The gene IDs for these targets were retrieved from UniProt. A protein–protein interaction network for the 63 nodes was generated using the STRING database. The network was built with default settings and a medium confidence score of 0.400. All available active interaction sources were included, and nodes without edges were removed for further analysis. Pathway enrichment analysis (PEA) was conducted using Reactome and KEGG pathways. The Gene Ontology (GO) enrichment analysis for molecular functions (MF), biological processes (BP), and cellular components (CC) of the gene sets was performed using the STRING web server.

### Histopathological evaluation

The specimens collected from the hippocampus and prefrontal cortex (N = 3) were fixed in 10% formalin for 24–48 h to harden them, prevent autolysis, preserve tissue structure, and prevent shrinkage. Tissue sections were de-paraffinized in xylene, cleaned with alcohol, stained with hematoxylin, counterstained with eosin, dehydrated, cleared with xylene, and mounted using Canada balsam. Neurodegeneration was measured using the semi-quantitative scoring method in the rat brain samples and were performed in a blinded manner.

### Statistical analysis

All the data are expressed as mean ± standard deviation (SD). Behavioral assessment including MWM and EPM (N = 6) were analyzed using one-way ANOVA, followed by the Bonferroni *post-hoc* test. Biomarkers (N = 6) were analyzed using one-way ANOVA followed by the Bonferroni post-hoc test in Graph Pad Prism 5.0 (La Jolla, CA, United States). Principal coordinates analysis was performed using QIIME2 software to observe the clustering pattern among samples. Microbial abundance across groups was analyzed using the non-parametric Kruskal–Wallis test, followed by Dunn’s multiple comparison test. Pearson’s correlation coefficient was used to assess the association between variables, such as Shannon index and AChE activity, using SPSS software (Version 21). The correlation coefficient (r) ranging from −1 to +1 indicates perfect negative to perfect positive correlation, respectively. Correlation strength was interpreted as weak (0–0.3), moderate (0.3–0.7), or strong (0.7–1.0). Statistical significance was set at *p* < 0.05.

## Results

### Behavioral assessment

**FIGURE 3:**
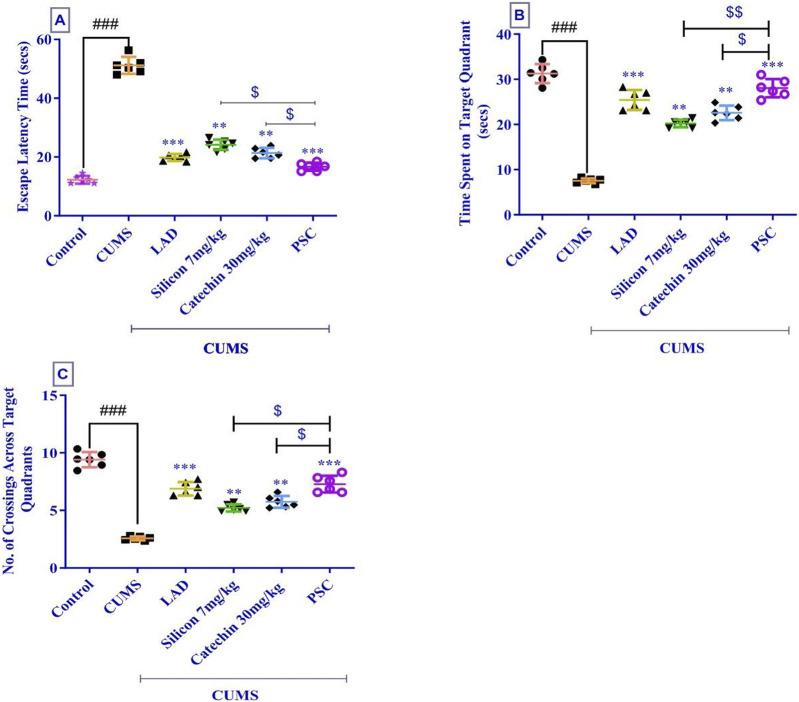
Effect of PSC on Morris Water Maze Experiment: **(A)** Escape latency time; **(B)** Time spent on target quadrant; **(C)** Number of crossings across target quadrant. Mean ± Standard deviation, N = 6. Interpretation done using one-way ANOVA followed by Bonferroni post hoc test. Significance range mentioned ^##^ *P* < 0.001 Vs Control. ***P < 0.001, **P < 0.01 Vs CUMS group. ^$^ *P* < 0.05, ^$$^ *P* < 0.01 Vs PSC.

In the MWM test (n = 6), CUMS showed increased Escape latency time (ELT) compared to that in the control [F (5, 30) = 11.01, *p* < 0.001]. Standard Lactobacillus acidophilus (LAD) [F (5,30) = 9.83, *p* < 0.001], silicon (7 mg/kg) [F (5,30) = 7.6, *p* < 0.01], catechin (30 mg/kg) [F (5,30) = 9.41, *p* < 0.01], and their combination PSC [F (5,30) = 9.74, *p* < 0.001] significantly reduced ELT compared to CUMS. CUMS decreased time spent in the target quadrant region [F (5,30) = 10.3, *p* < 0.001], while LAD [F (5,30) = 8.63, *p* < 0.01], silicon (7 mg/kg) [F (5,30) = 6.11, *p* < 0.01], catechin (30 mg/kg) [F (5,30) = 6.63, *p* < 0.01], and their combination PSC [F (5,30) = 7.81, *p* < 0.001] increased it significantly. Similarly, the number of crossings decreased in CUMS [F (5,30) = 7.1, *p* < 0.001] but increased with LAD [F (5,30) = 6.3, *p* < 0.01], silicon (7 mg/kg) [F (5,30) = 4.6, *p* < 0.01], catechin (30 mg/kg) [F (5,30) = 4.91, *p* < 0.01], and their combination PSC [F (5,30) = 5.8, *p* < 0.001]. Among the treatment group animals, PSC-treated animals showed better responses in the MWM test. Among the treatment group animals, PSC-treated animals showed [F (5,30) = 3.2, *p* < 0.05] significantly improved ELT, time spent in the target area, and the number of crossings compared to silicon- and catechin-alone-treated animals (Figures 3A–C).

**FIGURE 4:**
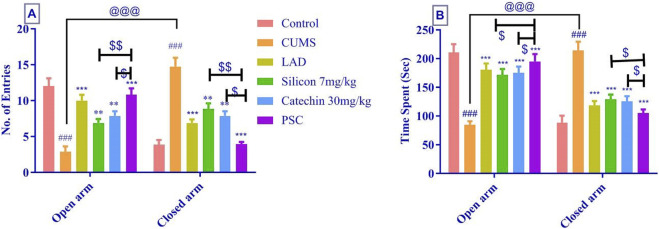
Effect of PSC on elevated plus maze: Mean ± Standard deviation, N = 6. **(A)** No of entries in both arms. **(B)** Time spent in both arms. Statistical interpretation done using one-way ANOVA followed by Bonferroni post hoc test. Significance range mentioned ^##^ *P* < 0.001 Vs Control. ***P < 0.001, **P < 0.01 Vs CUMS group. ^$^ *P* < 0.05, ^$$^ *P* < 0.01 Vs PSC. ^@@@^ *P* < 0.001 open arm Vs closed arm.

In the plus maze assessment (n = 6), the number of entries into the open arm was decreased significantly in the CUMS group compared to that in the control [F (5,30) = 8.2, *p* < 0.001]. However, LAD significantly increased the entries into the open arm compared to CUMS [F (5,30) = 7.32, *p* < 0.001]. Similarly, treatment with silicon (7 mg/kg) [F (5,30) = 7.6, *p* < 0.01], catechin (30 mg/kg) [F (5,30) = 6.2, *p* < 0.01], and their combination PSC [F (5,30) = 7.6, *p* < 0.001] significantly increased entries into the open arm compared to CUMS. Time spent in the open arm decreased significantly for the CUMS group compared to controls [F (5,30) = 5.8, *p* < 0.01]. However, LAD significantly increased the time spent in the open arm compared to CUMS [F (5,30) = 4.7, *p* < 0.001]. Similarly, treatment with silicon (7 mg/kg) [F (5,30) = 3.6, *p* < 0.001], catechin (30 mg/kg) [F (5,30) = 3.9, *p* < 0.001], and their combination of PSC significantly increased the time spent in the open arm compared to CUMS [F (5,30) = 4.3, *p* < 0.001]. Furthermore, compared to the catechin and silicon treatment group, PSC-treated animals showed [F (5,30) = 4.51, *p* < 0.05] a significant improvement in the number of entries and time spent in the open arms, indicating a primary anxiolytic-like response (Figures 4A,B).

### Acetyl cholinesterase activity

**FIGURE 5:**
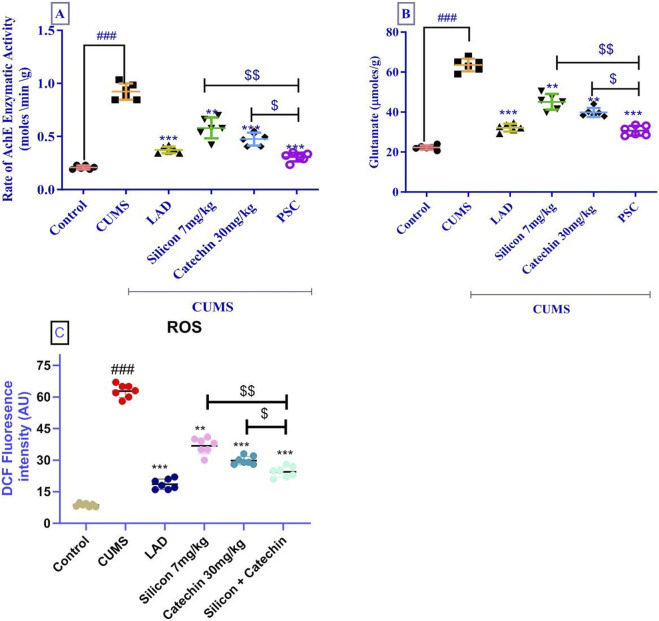
Effect of PSC on **(A)** Plasma Acetyl cholinesterase activity; **(B)** Glutamate release; **(C)** Brain ROS assay. Mean ± Standard deviation, N = 6. Statistical interpretation done using one-way ANOVA followed by Bonferroni post hoc test. Significance range mentioned ^##^ *P* < 0.001 Vs Control. ***P < 0.001, **P < 0.01 Vs CUMS group. ^$^ *P* < 0.05, ^$$^ *P* < 0.01 Vs PSC.

Compared to the control group (n = 6), the CUMS group showed a significant increase in acetylcholinesterase enzyme activity [F (5, 30) = 12.23, *p* < 0.001]. LAD [F (5, 30) = 9.3, *p* < 0.01] decreased the rate of enzyme activity compared to the CUMS group. Treatment with silicon at 7 mg/kg [F (5,30) = 5.4, *p* < 0.01], catechin at 30 mg/kg [F (5,30) = 6.21, *p* < 0.001], and combination of PSC [F (5,30) = 7.11, *p* < 0.001] significantly decreased the rate of enzyme activity compared to the CUMS group. However, compared to the catechin treatment group, PSC-treated animals showed [F (5,30) = 4.21, *p* < 0.05] significantly decreased cholinesterase enzyme activity (Figure 5A).

### Effect of silicon-mixed catechin on the glutamate level

Compared to the control group (n = 6), the CUMS group significantly increased the plasma glutamate level [F (5, 30) = 15.13, *p* < 0.001]. In contrast, LAD [F (5, 30) = 13.26, *p* < 0.001] decreased the plasma glutamate level compared to the CUMS group. Treatment with silicon at 7 mg/kg [F (5, 30) = 11.35, *p* < 0.01] and catechin at 30 mg/kg [F (5, 30) = 10.11, *p* < 0.01] significantly decreased the glutamate level compared to the CUMS group. Combination of PSC [F (5, 30) = 13.9, *p* < 0.001] also decreased the plasma glutamate level significantly compared with the CUMS group. Moreover, compared to the catechin treatment group, PSC-treated animals showed [F (5, 30) = 3.82, *p* < 0.05] a significant decrease in the plasma glutamate level (Figure 5B).

### Effect of silicon-mixed catechin on the brain ROS level

The CUMS group showed a considerable increase in the DCF fluorescence intensity range (n = 6) [F (5, 30) = 30.12, *p* < 0.001] compared to the control group. However, LAD [F (5, 30) = 25.23, *p* < 0.001] displayed a decreased fluorescence intensity range compared to the CUMS group. Treatment with silicon at 7 mg/kg [F (5, 30) = 16.25, *p* < 0.01] and catechin at 30 mg/kg [F (5, 30) = 19.61, *p* < 0.01] showed a decrease in the DCF fluorescence intensity range compared to the CUMS group. Combination of PSC [F (5, 30) = 21.41, *p* < 0.001] displayed an extensively decreased fluorescence intensity range compared with the CUMS group and other treatment groups (Figure 5C).

### Effect of silicon-mixed catechin on the antioxidant enzyme level

**FIGURE 6:**
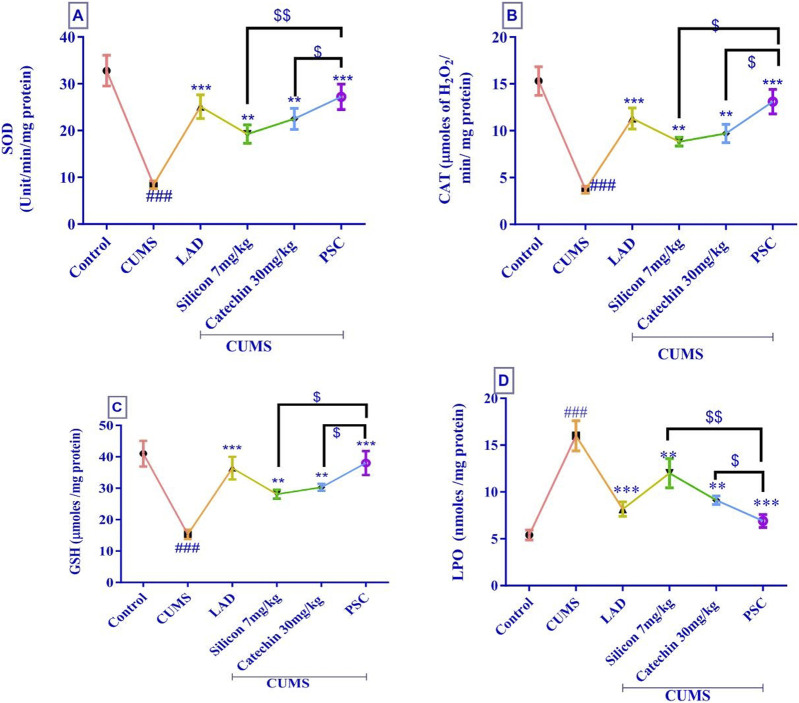
Effect of PSC on brain anti-oxidant enzyme levels. **(A)** SOD; **(B)** CAT; **(C)** GSH; **(D)** LPO. Mean ± Standard deviation, N = 6. Statistical interpretation done using one-way ANOVA followed by Bonferroni post hoc test. Significance range mentioned ^##^ *P* < 0.001 Vs control. ***P < 0.001, **P < 0.01 Vs CUMS group. ^$^ *P* < 0.05, ^$$^ *P* < 0.01 Vs PSC.

When compared to the control group (n = 6), the CUMS group exhibited a significant decrease in SOD, CAT, and GSH levels, along with an increase in LPO levels (*p* < 0.001) (Figures 6A–D). However, LAD significantly increased SOD (p < 0.001), CAT (*p* < 0.001), and GSH levels (*p* < 0.001) while decreasing LPO levels (*p* < 0.001) compared to the CUMS group. Similarly, treatment with silicon (7 mg/kg) (*p* < 0.01), catechin (30 mg/kg) (*p* < 0.01), and their combination (PSC) significantly increased SOD, CAT, and GSH levels while decreasing LPO levels compared to the CUMS group (*p* < 0.001). Among treatment groups, the level of antioxidant markers SOD, CAT, GSH, and LPO were significantly reversed by the PSC treatment [F (5, 30) = 4.14, *p* < 0.05] compared to the catechin-treated rats.

### 16S rRNA V3V4 metagenomics analysis

**FIGURE 7:**
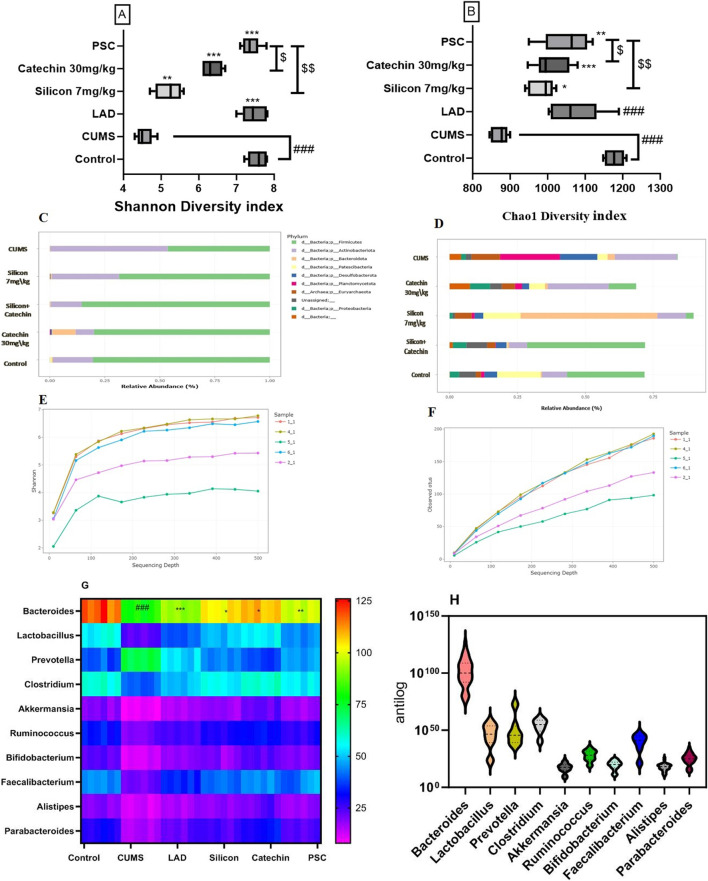
16S rRNA V3V4 metagenomics analysis. **(A)** Shannon index; **(B)** Chao1 diversity index; **(C)** phylum-level taxonomy; **(D)** genus-level taxonomy; **(E,F)** alpha rarefaction curve. **(G)** Heat map shows the significant bacterial taxa association with different treatment groups. Scale range indicates that blue color represents the negative relationship and red color indicates a positive relationship of bacterial taxa. **(H)** Overall abundance of specific bacterial phyla using the PCoA plot beta-diversity index. Mean ± standard deviation, (N = 6). Microbial abundance was assessed using the non-parametric Kruskal–Wallis test followed by Dunn’s multiple comparison test. Significance range mentioned ^##^ *p* < 0.001 vs. control. ***p < 0.001 and **p < 0.01 vs. CUMS group. ^$^ *p* < 0.05 and ^$$^ *p* < 0.01 vs. PSC.

The Chao1 diversity index and the Shannon diversity index were inferred based on the assessment of microbial species in the treated and untreated groups (n = 6) (Figures 7A–F). The Shannon index measures α-diversity in terms of total taxa and their relative abundances, and the Chao1 index measures the estimated richness of a sample. CUMS displayed a significant reduction in the Chao1 diversity index (885.8) compared to the catechin-treated group (1,175.64) and the PSC-treated group (1,059.41). On the other hand, the Shannon diversity index was used to calculate the relative abundance of microbial species. Rats with chronic stress exhibited a significant reduction in the Shannon diversity index (5.9) compared to the catechin-treated group (7.33) and the PSC group (7.52). This indicates an improvement in relative abundance within the microbial community. Furthermore, when compared to the catechin-treated group, PSC-treated animals [F (5, 30) = 3.21, *p* < 0.05] significantly improved the Chao1 diversity index and the Shannon diversity index, indicating the effectiveness of combination therapy.

**FIGURE 8:**
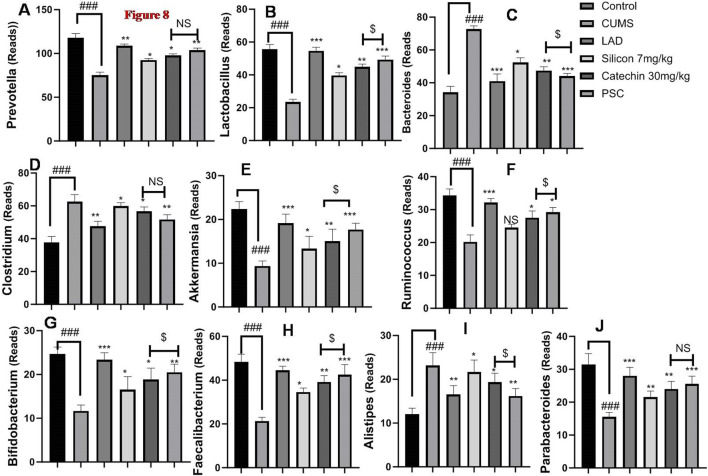
Represent the specific bacterial taxa such as **(A)** *Prevotella*; **(B)** *Lactobacillus*; **(C)** *Bacteroides*; **(D)** *Clostridium*; **(E)** *Akkermansia*; **(F)** *Ruminococcus*; **(G)** *Bifidobacterium*; **(H)** *Faecalibacterium*; **(I)** *Alistipes*; **(J)** *Parabacteroides* of gut microbiome in CUMS induced rats (n=6). Statistical interpretation done using one-way ANOVA followed by Bonferroni post hoc test. Significance range mentioned *^##^* *P* < 0.001 Vs control. ***P < 0.001, **P < 0.01 Vs CUMS group. ^$^ *P* < 0.05, ^$$^ *P* < 0.01 Vs PSC.

We observed the taxonomical alteration after the treatment against stress condition and demonstrated through heat map analysis and the β-diversity index (Figures 7G,H). Chronic stress positively associates with certain bacterial phyla such as *Bacteroides*, *Clostridium*, and *Alistipes*, while it negatively associates with *Prevotella*, *Lactobacillus*, *Akkermansia*, *Ruminococcus*, *Bifidobacterium*, *Faecalibacterium*, and *Parabacteroides.* Subsequently, treatment with silicon, catechin, and PSC significantly increased the abundance of bacterial phyla. *Bacteroides* [F (5, 30) = 14.31, *p* < 0.001] were the maximum, followed by the *Clostridium* [F (5, 30) = 10.43, *p* < 0.01] and *Alistipes* [F (5, 30) = 11.13, *p* < 0.01], compared to the CUMS group. Similarly, treatment with silicon, catechin, and PSC significantly decreased the abundance of *Prevotella* [F (5, 30) = 8.26, *p* < 0.01], *Lactobacillus* [F (5, 30) = 21.44, *p* < 0.001], *Akkermansia* [F (5, 30) = 24.50, *p* < 0.05], *Ruminococcus* [F (5, 30) = 7.42, *p* < 0.05], *Bifidobacterium* [F (5, 30) = 14.11, *p* < 0.01], Faecalibacterium [F (5, 30) = 18.71, *p* < 0.001], and *Parabacteroides* [F (5, 30) = 20.38, *p* < 0.001] compared to the CUMS group. On the other hand, comparison of PSC and silicon and catechin displayed significant improvement in *Lactobacillus*, *Akkermansia*, *Ruminococcus*, *Bifidobacterium*, and *Faecalibacterium*, but not in *Prevotella*, *Clostridium*, and *Parabacteroides* (Figure 8).

### Functional pathway prediction analysis

**FIGURE 9:**
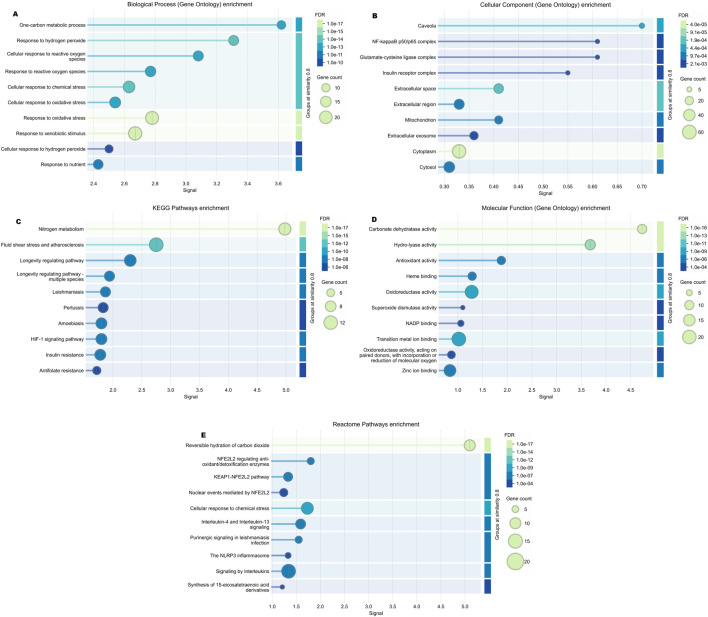
Pathway prediction analysis: mechanism of catechin in dementia anticipated using network pharmacology. KEGG pathway prediction analysis computed using the FDR range. Signal or enrichment score mentioned in X-axis indicates strength of enrichment, bubble size represents the number of genes associated, and color depth indicates the significance range; **(A)** enrichment of biological processes of the 10 common genes; **(B)** enrichment of cellular response of the 10 common genes; **(C)** top enriched pathways of the 10 common genes; **(D)** enrichment of molecular functions of the 10 common genes; **(E)** reactome pathway enrichment of the common genes.

A total of 63 gene targets were identified for the catechin molecule. The top 10 biological responses predicted and listed for the catechin molecule based on the significance range included one-carbon metabolic process, response to hydrogen peroxide, cellular response to reactive oxygen species, and response to oxidative stress, indicating a significant association with hydrogen peroxide and reactive oxygen species (ROS) pathways involved in the regulation of oxidative stress. The top 10 cellular protein components for catechin based on the significance range included caveolae, NF-κB complex, mitochondrion, and glutamate cysteine ligase complex, indicating a strong association with cellular energy metabolism, ROS redox balance, and antioxidant defense mechanisms. Top 10 KEGG signaling pathways predicted for the catechin molecule included nitrogen metabolism, fluid shear stress, and atherosclerosis and longevity-regulating pathways, indicating an association with metabolic adaptation, aging regulation, and stress signaling pathways. The top 10 molecular functional pathways associated with catechin included carbonate dehydratase activity, hydrolase activity, and antioxidant activity, merely connecting with redox regulation. The top 10 specific molecular pathways were predicted in the Reactome database, such as reversible hydration of carbon dioxide, NRF2-regulating antioxidant detoxification enzymes, and KEAP1–NRF2 pathway, indicating a strong association with antioxidant defense mechanisms and inflammatory pathways (Figure 9).

### Brain histology

**FIGURE 10:**
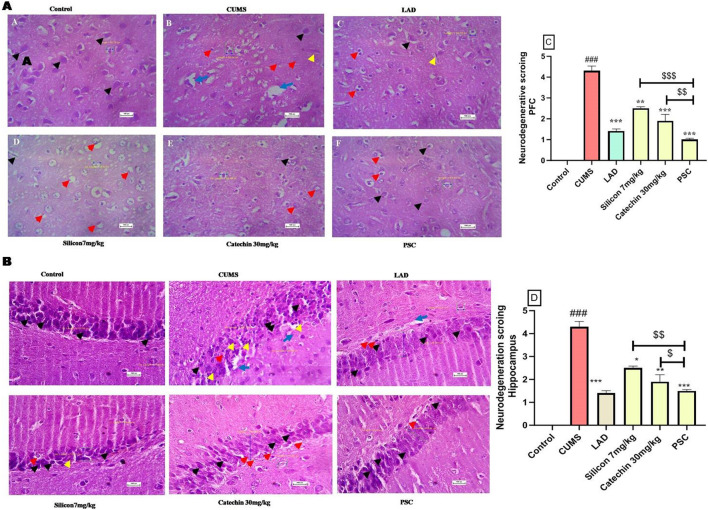
Histopathology of the hippocampus and prefrontal cortex region, with hematoxylin and eosin staining magnified at ×40 (N = 3). Prefrontal cortex **(A,C)** and hippocampus **(B,D)**. The black arrow represents normal neuronal cells with nucleus and Nissl substances; the red arrow represents a fragmented nucleus or a pyknotic nucleus; the blue arrow indicates edematous tissue; the yellow arrow represents neuronal degeneration. Scoring pattern; 0—normal histology; 1—mild neuronal damage (few pyknotic cells); 2—moderate neuronal damage (obvious neuronal loss and vacuolation); 3—severe neuronal damage (excessive gliosis); 4—very severe neuronal damage (extensive neuronal loss).

Histopathological scores were used to assess neurodegeneration in the rat brain samples (n = 3). CUMS rats exhibited significant neurodegeneration compared to the control group [F (2, 18) = 21.08, *p* < 0.001]. In contrast, LAD remarkably decreased neurodegeneration compared to the CUMS [F (2, 18) = 18.08, *p* < 0.001]. When compared to the CUMS, catechin, silicon, and PSC treatment remarkably decreased neurodegeneration [F (2, 18) = 11.21, *p* < 0.001, F (2, 18) = 9.32, *p* < 0.01 and F (2, 18) = 17.51, *p* < 0.001]. Among treatment groups, neurodegeneration was significantly reversed by the PSC treatment [F (2, 18) = 1.51, *p* < 0.01] compared to the catechin-treated animals (Figure 10).

### Correlation study

**FIGURE 11:**
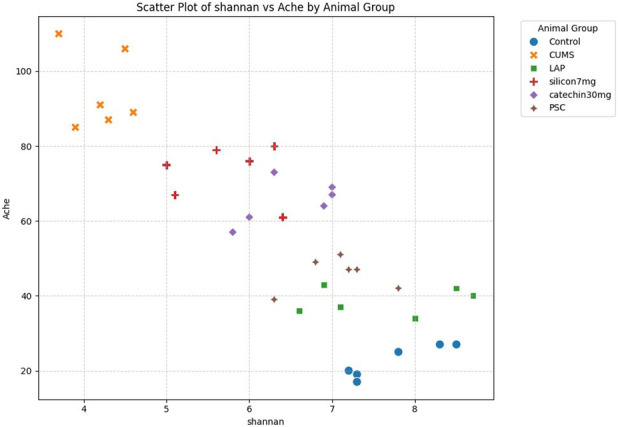
Correlation analysis: Pearson correlation coefficient (r) analysis performed between variables (n = 6), Figure depicts the scattered plot of the Shannon index and AchE activity.

The Pearson correlation study represents alliance of the Shannon index with AChE enzyme activity (Figure 11). In the CUMS group, a statistically significant strong negative correlation was observed (r = −0.930, *p* = 0.007). In LAP, a statistically significant moderate negative correlation was observed (r = −0.695 p = 0.012). Silicon 7 mg/kg showed no correlation (r = −0.036, *p* = 0.146). Catechin 30 mg/kg showed a statistically significant weak negative correlation (r = −0.211, *p* = 0.039). PSC showed a statistically significant moderate negative correlation (r = −0.511, *p* = 0.012). This indicates that the Shannon index and AChE enzyme activity are correlated, and the data show that when the Shannon index increases, AChE enzyme activity decreases, and *vice versa*.

## Discussion

Acute and chronic stress negatively impact health by altering the composition of the gut bacteria in various gut regions, including the lumen and the mucosal lining. Chronic stress can significantly disrupt the structural organization of gut microbes, potentially affecting intestinal metabolites and hormonal fluctuations, which may lead to more sustained and profound negative outcomes that closely resemble pathological features observed in Alzheimer’s disease and dementia (Cruz-Pereira et al., 2020; Gao et al., 2018). There is a bidirectional relationship between gut microbiota alterations and neurodegenerative diseases such as Alzheimer’s disease (Zhu et al., 2021). Several studies demonstrate a direct connection between gut function and neuropsychological conditions including depression, anxiety, and mood-related disorders (Fond et al., 2014). Hence, we investigated how continuous unpredictable mild stress over 28 days impacts the gut microbiome and contributes to dementia. Previous studies have reported that 20–75 mg/day dietary silicon intake is safe in humans as soluble silicon is highly excreted from the body and does not produce toxicity (Melenikioti et al., 2026). The average silicon intake in rats through drinking water is 0.37 mg/100 gm of body weight, which is considered safe, and doses up to 10 mg/kg did not show major hepatic or renal toxicity (Wu et al., 2021). A moderate dose of silicon supports mucosal membrane integrity and helps adapt microbial composition without provoking dysbiosis (Hernández-Martín et al., 2025). Accordingly, in this study, 7 mg/kg dose of silicon was selected based on its safety profile and microbiome-modulating potential. (+)/−catechin was administered at doses between 25 mg and 100 mg/kg (28 days orally) in rats, relevant for antioxidant/oxidative stress outcomes (Del Carmen García-Rodríguez and Kacew, 2025). Accordingly, a dose of 30 mg/kg catechin was chosen as a moderate and pharmacologically relevant intervention. The rationale for combining porous silicon water with catechin was based on the possibility that this formulation could simultaneously target oxidative stress and gut microbial imbalance under chronic stress conditions.

The MWM and EPM are essential tools in behavioral neuroscience research, offering insights into stress-mediated spatial learning, retrieval memory, and fear-conditioning responses in rodents (Othman et al., 2022). The MWM evaluates hippocampal-dependent spatial learning and reference memory ability. Measuring ELT reflects the capacity to learn and remember the hidden platform location representing spatial navigation and memory consolidation (Tucker et al., 2018). Rats treated with PSC exhibited shorter escape latencies, indicating improved memory consolidation. Liang et al. (2024) stated that spending more time in and crossing the target quadrant, where the hidden platform was located, indicates stronger spatial learning and memory retrieval. In parallel, PSC-treated animals displayed significant open-arm exploration in the EPM, suggesting the attenuation of stress-induced anxiety-like behavior. Such behavioral improvement may be functionally linked to preserved synaptic plasticity in hippocampal subregions implicated in learning and memory, including CA1 and CA3, as previously reported for anxiolytic and neuroprotective interventions (Moreno-Martínez et al., 2022). Taken together, these data indicate that PSC effectively ameliorates both cognitive and affective disturbances induced by chronic stress.

Dysregulation of cholinergic neurons is critical to dementia and other neurodegenerative conditions (Pepeu and Grazia Giovannini, 2017). Chronic stress causes cerebral atrophy and accelerates choline acetyltransferase enzyme activity. It also catalyzes the hydrolysis of acetylcholine at the synapse, promoting immediate clearing of acetylcholine and leading to impaired cholinergic regulation (Pegueroles et al., 2018). We demonstrate that PSC treatment decreases the acetylcholinesterase enzyme activity, thereby contributing to spatial learning and memory improvement.

Glutamate regulators positively support neuroplasticity (Pal, 2021) and are exceptionally important in mood and cognitive function (Dauvermann et al., 2017). Maintaining optimal levels of glutamate is essential as low levels can deplete energy, while elevated levels can lead to cell death (Zhou and Danbolt, 2014). However, chronic stress increases the glutamate release in the hippocampus and decreases in the prefrontal cortex and amygdala by altering neuronal glutamine synthase activity. This leads to learning and memory impairment, attention deficit, and anxiety-like behavior (Pal, 2021; Popoli et al., 2011). In the present study, PSC treatment significantly reduced plasma glutamate levels in stressed animals, consistent with an attenuation of excitotoxic burden. This observation aligns with previous reports demonstrating that catechin-related polyphenols, including epigallocatechin gallate (EGCG), can improve cognitive performance by modulating glutamatergic neurotransmission and limiting oxidative damage (Schaffer et al., 2012). Thus, the glutamate-lowering effect of PSC may represent an additional pathway through which this intervention delays the progression of dementia.

Chronic stress vulnerably affects the hippocampus and prefrontal cortex by altering the antioxidant defense mechanism. It has been observed that induction of chronic stress causes major cell injury provoked by the lipid peroxidation in the cell membrane due to the release of reactive oxygen species (Herbet et al., 2017). Oxidative damage in the cell is induced by the ROS system, including hydroxyl radicals, hydrogen peroxide, and superoxide ions, which are highly reactive and chemically unstable. The cellular redox mechanism is highly disturbed by the CUMS, promoting reactive oxygen species through the HPA axis (Samarghandian et al., 2017). Antioxidants restore cellular damage by donating or transferring hydrogen atoms to unstable reactive molecules, converting them into stable non-reactive substances (Lobo et al., 2010). In our study, PSC treatment significantly reduced the H_2_O_2_-initiated DCF fluorescence intensity in the CUMS rats, indicating a lower level of unstable reactive oxygen species in brain tissues. Notably, these effects were more pronounced than those observed with catechin alone, suggesting a possible synergistic advantage of the combined formulation.

In the present study, continuous exposure to different stressful stimuli significantly increased the lipid peroxidation and reduced the antioxidant enzymes such as SOD, CAT, and GSH, confirming that CUMS triggers free radical generation in the hippocampus and prefrontal cortex (Nimetİzgüt-Uysal et al., 2004). PSC administration exhibited elevated levels of SOD and CAT in brain tissues, along with increased glutathione levels and decreased lipid peroxidation, indicating a balancing effect on the ROS system. Even though catechin is a very good antioxidant, gut microbes did not receive much benefit under stressful conditions due to the reduced number of beneficial bacterial colonies. We describe that the antioxidant effect of catechin is improved when combined with porous silicon water, so that the abundance of beneficial bacteria was increased significantly compared to that in catechin-treated rats, as evidenced by the *post-hoc* test. This observation is further supported by an earlier study which reports that the silicon molecule releases additional hydrogen ions when mixed with water, as described using the following equation: Si + 2H_2_O → SiO_2_ + 2H_2_ (Imamura et al., 2017).

Dementia has been linked to changes in gut microbiome composition, such as low short-chain fatty acids that impair microglial activity, leading to neuroinflammation, which, in turn, contributes to the buildup and poor removal of amyloid beta in the brain (Giau et al., 2018; Mukherjee and Reddy, 2025). A study reported a significant disparity between gut microbiome and brain function in stressed rats (O’Riordan et al., 2022). With this note, the relative abundance of bacteria at the phylum and genus levels was studied using 16S mRNA sequencing. At the phylum scale, stressed rats showed reduced levels of *Firmicutes* and *Bifidobacterium* and increased levels of *Bacteroidetes*, which is consistent with human studies on the stool of Alzheimer’s patients (Chandra et al., 2023). Conversely, the PSC group exhibited elevated levels of *Firmicutes* in the gut. In this study, the top 10 abundant genera were *Lactobacillus*, *Collinsella*, *Streptococcus*, *Romboutsia*, *Allobaculum*, *Rothia*, *Holdemanella*, *Blautia*, and *Catenibacterium*. Among this, relative abundance of *Lactobacillus* was significantly higher in rats treated with PSC (43.45%) and catechin (10.07%) compared to the CUMS rats (0.25%). At the genus level, CUMS exhibited *Lactobacillus* 4%, *Bifidobacterium* 2%, and *Escherichia* 12%, but PSC treatment resulted in *Lactobacillus* 15% (3.2-fold increase), *Bifidobacterium* 10% (4.2-fold increase), and *Escherichia* 5% (39-fold decrease). At the species level, CUMS rats exhibited *Lactobacillus rhamnosus* 1.7% and *Escherichia coli* 11%, whereas PSC treatment exhibited *Lactobacillus rhamnosus* 7% and *Escherichia coli* 5%. The pronounced increase in *Lactobacillus* abundance following PSC administration, together with the reduction in *Escherichia coli,* further supports a stress-resilient microbial reconfiguration. These findings suggest that PSC treatment may support the gut microbial composition by promoting a more balanced and functionally favorable bacterial community.

Chronic stress has also been linked to long-term alterations in the gut microbiome, leading to variations in α- and β-diversity of gut bacteria, as evident from the Shannon index and Chao index. These metrics were utilized to evaluate the biodiversity of the gut microbiota. The α-diversity offers a summary of the microbial community and its balance, whereas the β-diversity offers dissimilarity of species or richness (Bartha et al., 2024). The present study aligns with these indices, indicating that CUMS-induced reduction in the Chao1 diversity index and the Shannon diversity index were reversed after PSC treatment, creating a balanced gut biodiversity environment. In addition to that, we advocate that modulation in relative abundance of bacterial phylum and genus level is strongly associated with learning and memory processes.

PSC treatment remarkably reduced the abundance of *Bacteroides, Clostridium*, and *Alistipes* during stress. This is supported by a previous study, which states that *Bacteroides*, a Gram-negative bacterial genus, is significantly associated with dementia and anxiety-related disorders. *Clostridium* is excessively produced in Alzheimer’s condition, and *Alistipes*, highly influenced by stress, affects mood and inflammation (Bonnechère et al., 2022; Kaiyrlykyzy et al., 2022; Tana et al., 2025). In addition to this, certain gut microbes were significantly increased after PSC treatment, including *Lactobacillus*, *Prevotella*, *Akkermansia*, *Ruminococcus*, *Bifidobacterium*, *Faecalibacterium*, and *Parabacteroides.* These findings are supported by earlier studies stating that *Lactobacillus* is a beneficial probiotic releasing bacteria and is highly suppressed in neurological disorders affecting cognitive function. *Prevotella* is a short-chain fatty acid producing bacteria and is positively correlated with anti-inflammatory and microglial maturation processes. *Akkermansia* is a mucin-degrading bacterium and supports intestinal permeability and improves behavioral outcomes under stress. *Ruminococcus* is a fiber-fermenting bacterium, which is involved in metabolic regulation in the gut and negatively associated with dementia. *Bifidobacterium* is a probiotic-producing bacterium, which regulates GABA transmission and folate metabolism in Alzheimer’s disease. A reduced level of *Faecalibacterium* is consistently reported in mild cognitive decline and dementia condition. *Parabacteroides* significantly alter the bile acid metabolism in the gut and have been implicated in various neurological conditions (Ding et al., 2021; Kaiyrlykyzy et al., 2022). Together, these findings suggest that PSC treatment favorably modulates the gut microbiome by promoting beneficial bacterial populations in the CUMS-induced dementia model.

Stress can also influence the functional aspects of the hippocampus and prefrontal cortex, which are crucial for spatial navigation and memory. Anatomically, stress is often associated with reduced hippocampal volume, abnormal glial cells, impaired neurogenesis, and dendritic atrophy (Kim et al., 2015). In the present study, CUMS-induced rat brains depict remarkable neurodegenerative changes, such as gliosis, lymphocytic infiltration, neuronal cell vacuolation, and inflammation in the prefrontal region. Furthermore, hippocampal cell shrinkage, fewer degenerative pyramidal cells in the cerebellar area, and prominent cell necrosis were also observed in the CUMS brain. Treatment with PSC showed mild edema with discrete pyramidal cells, and the hippocampal cells appeared relatively normal with mild inflammation and no gliosis. No acute necrotic cells were found in the cortex and hippocampus region.

Collectively, the present findings identify PSC intervention as capable of attenuating chronic stress-induced cognitive deficits through associated modulation of oxidative stress, cholinergic and glutamatergic signaling, gut microbial composition, and neuropathological integrity. Although additional mechanistic studies are warranted to define causal pathways more precisely, the study suggests that porous silicon water combined with catechin treatment may significantly contribute to improving the chronic unpredictable mild stress-induced dementia-like conditions in rats.

## Data Availability

The datasets presented in this study can be found in online repositories. The names of the repository/repositories and accession number(s) can be found in the article/supplementary material. SRA records are accessible through the following Gen-bank link under accession number PRJNA1469811: NCBI SRA PRJNA1469811.
